# Tailoring Nonlinear Metamaterials for the Controlling of Spatial Quantum Entanglement

**DOI:** 10.3390/nano12224001

**Published:** 2022-11-13

**Authors:** Yang Ming, Yuan Liu, Wei Chen, Yusen Yan, Huiguo Zhang

**Affiliations:** 1 School of Electronic and Information Engineering, Changshu Institute of Technology, Suzhou 215000, China; 2 National Laboratory of Solid State Microstructures, College of Engineering and Applied Sciences, Nanjing University, Nanjing 210093, China

**Keywords:** nonlinear metamaterials, photonic entanglement, orbital angular momentum

## Abstract

The high designability of metamaterials has made them an attractive platform for devising novel optoelectronic devices. The demonstration of nonlinear metamaterials further indicates their potential in developing quantum applications. Here, we investigate designing nonlinear metamaterials consisting of the 3-fold (C3) rotationally symmetrical nanoantennas for generating and modulating entangled photons in the spatial degrees of freedom. Through tailoring the geometry and orientation of the nanoantennas, the parametric down conversion process inside the metamaterials can be locally engineered to generate entangled states with desired spatial properties. As the orbital angular momentum (OAM) states are valuable for enhancing the data capacity of quantum information systems, the photonic OAM entanglement is practically considered. With suitable nanostructure design, the generation of OAM entangled states is shown to be effectively realized in the discussed nonlinear metamaterial system. The nonlinear metamaterials present a perspective to provide a flexible platform for quantum photonic applications.

## 1. Introduction

Metamaterials are artificial composite structures with exotic electromagnetic properties and functionalities that cannot be obtained with naturally existing materials. The substructures of metamaterials can be flexibly designed, which endows them with unique advantages for devising novel functional optoelectronic devices. The current mature nanofabrication technologies provide powerful bases for the processing of metamaterials [1], leading to the development of metamaterial devices in the classical realm, including metalenses [2], singular beam generators [3,4] and perfect absorber [5]. However, the potential of metamaterials in the quantum field has not been adequately realized.

In this work, we are devoted to developing metamaterial design for tailoring quantum entanglement. The entangled states are particularly useful for a series of quantum information applications, including quantum cryptography [6], quantum computation [7] and quantum metrology [8]. Parametric down conversion (PDC) is a well-established and widely used method for generating entangled photons [9]. Traditionally, nonlinear optical crystals have been the most popular experimental twin-photon source, such as lithium niobate (LN) [10] and barium borate (BBO) [11]. However, along with the development of quantum photonic technologies, it faces the requirement of practicality, thus the capacity of tailoring the properties of entangled photons is desired [12,13]. This task is inconvenient for nonlinear crystals due to the fixity of microstructures. Given that, we investigate generating and controlling photonic entanglement based on the parametric process in nonlinear metamaterials consisting of the 3-fold (C3) rotationally symmetrical nanoantennas. Compared with the previously proposed metamaterials based on split ring resonators (SRRs) [14], the C3 nanoantenna system here provides a more flexible platform, which can be used for simultaneous controlling of spin and orbital angular momentum. Through tailoring the nanostructures of nonlinear metamaterials, we can control the PDC process inside the medium to obtain target photon states. The entangled states based on the spatial degrees of freedom are considered because they are of great value for enhancing the data capacity of quantum information systems [15]. With a suitable design, the generation of two-photon orbital angular momentum (OAM) entanglement is proposed. The lithium niobate and semiconductor metasurfaces have been demonstrated [16,17], but the spatial quantum entanglement is not considered. The OAM degree of freedom can be used for the construction of high dimensional Hilbert space, which is particularly valuable for quantum information applications.

## 2. Materials and Methods

The recent progress of nonlinear metamaterial fabrication provides practical bases for its quantum applications [18]. Compared to traditional nonlinear optical crystals, diverse nanostructures of the metamaterials endow the corresponding systems with excellently tunable features. Though the inner structures of some ferroelectric crystals, such as lithium niobate and lithium tantalite can be engineered based on the periodic poling technology [12,19], the micro-scale manufacture precision restricts the modulation accuracy and makes it lower than that of the metamaterial systems. This advantage allows nonlinear metamaterials to become an ideal platform for effective local controlling of light–matter interaction. Thus, the corresponding Hamiltonian interaction turns out to be space-dependent according to the nanostructures. Therefore, Hamiltonian engineering can be realized through nanostructure design. Based on this methodology, the generation dynamics of entangled photon states can be managed on demand to obtain desired spatial properties.

The actual compositions of structural units of metamaterials are manifold, but only a portion of them presents available nonlinearity, such as SRR [14,20] and nanoantenna with multi-rotational symmetry [21,22]. Here, we consider the meta-atom array system consisting of rotational symmetric nanoantennas [21,22]. Compared with SRR [14], such systems could be used for effective simultaneous tailoring of spin and orbital angular momentum through nonlinear optical interactions. In view of the structural complexity, a feasible approach to constructing a 3D system is stacking 2D metamaterial slices [23]. To describe the mechanisms of PDC in such a system, the whole medium can be divided into a series of differential elements along the direction perpendicular to the slices. Naturally, a 2D slice plays as a *dz*-element. In each element, the local PDC is actually lossy owing to the metallic compositions of the metamaterial [24,25]. A proper method should be proposed to deal with the issue of attenuation. There are various methods to deal with attenuation in quantum systems, including the Liouvillian formulation [26], the Green function [27] and the equivalent beam splitter model [28,29]. Here, we choose the beam splitter model according to the geometric character of the stacking system. We consider compartmentalizing the lossy PDC into two cascaded sub-processes. For the first sub-process, the decay is separated from the PDC and a local interaction Hamiltonian can be defined for this sub-process. For the second sub-process, the attenuation is equivalent as a beam splitter transformation [28,29].

To derive the interaction Hamiltonian of the first sub-process, we introduce the nonlinear Huygens–Fresnel principle [14,30]. In this theory, each point on the wavefront of the pump light serves as a sub-source of secondary down-converting waves through light-matter interaction. The effect of a point source can be expressed by the Hamiltonian density as:

(1) $$\begin{array}{cc} {ℋ}_{I}(\vec{r},\;t) & =\frac{1}{2}{\hat{P}}_{NM}(\vec{r},\;t)\cdot {\hat{E}}_{p}(\vec{r},\;t)\\ & =\frac{1}{2}\chi_{NM}^{\left(2\right)}(\vec{r}){\hat{E}}_{p}^{\left(+\right)}(\vec{r},\;t){\hat{E}}_{s}^{\left(-\right)}(\vec{r},\;t){\hat{E}}_{i}^{\left(-\right)}(\vec{r},\;t) \end{array}$$

In the above equation, *p*, *s* and *i* correspond to the pump, signal and idler photons, respectively. The parameter $\chi_{NM}^{\left(2\right)}$ is the second order nonlinear coefficient of metamaterials. For the electric fields, *E_p_* is usually treated to be classical, while *E_s_* and *E_i_* should be quantized with the creation and annihilation operators $a_{s(i)}^{†}$ and $a_{s(i)}^{}$.

To analyze the effect of intrinsic loss, the equivalent Hamiltonian of the beam splitter transformation is written as [14,28,31]:

(2) $$\begin{array}{cc} H_{BS}(z) & =\sum_{\sigma }\int d\omega_{s}\sqrt{2\gamma_{s}}\left[a_{s\sigma }b_{s\sigma }^{†}(z)+a_{s\sigma }^{†}b_{s\sigma }(z)\right]\\ & +\sum_{\sigma^{\prime }}\int d\omega_{i}\sqrt{2\gamma_{i}}\left[a_{i\sigma^{\prime }}b_{i\sigma^{\prime }}^{†}(z)+a_{i\sigma^{\prime }}^{†}b_{i\sigma^{\prime }}(z)\right] \end{array}$$

In this equation, the parameter *γ_s_*_(*i*)_ is the attenuation coefficient, while the operator $b_{s(i)}^{†}(z)$ describes that a photon gets into the loss channel at coordinate *z*.

**Figure 1:**
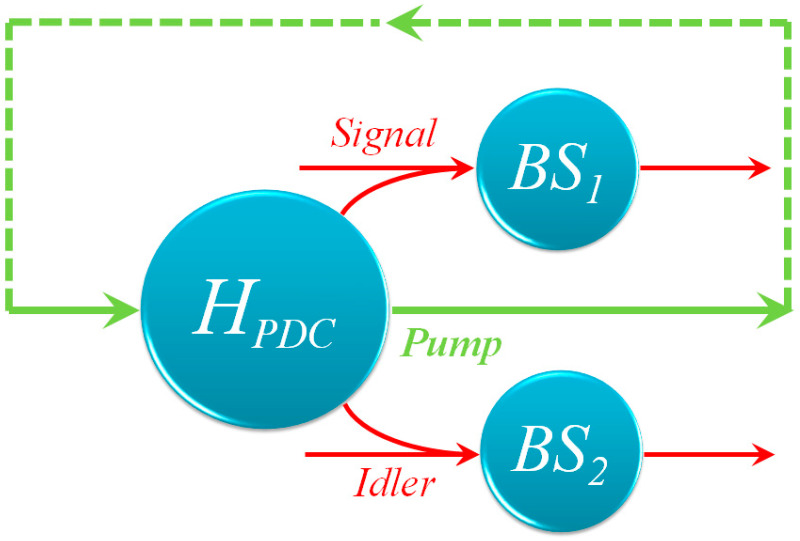
The illustration of a *dz*-cell. The local Hamiltonian describes the interaction of pump, signal and idler photons in nonlinear metamaterials, while the loss is represented by beam splitter transformation (*BS_1_* and *BS_2_*).

For the validity of this method, the system damping should be relatively low, thus it is required that the meta-atom is small and the corresponding array is not dense. The form of the final state can be obtained by combining all the *dz*-elements in sequence. The corresponding illustration is shown in Figure 1.

## 3. Results

Based on the proposed method, entangled photon states with any desired spatial properties can be realized through suitable design in theory. Here, we consider the generation of OAM entangled state. The OAM of a photon is associated with its wavefront. Correspondingly, the light beam carrying OAM is usually known as optical vortex with the helical phase *exp*(*ilφ*) [32], where *m* is the quantum number of OAM defined as the topological charge. This suggests that the quantity of OAM with each photon is *lℏ*. As a high-dimensional Hilbert space can be constructed based on the OAM degree of freedom, it is extremely valuable for expanding information capacity [15,33], hence the OAM entangled state can be extremely useful in quantum information processing. The metamaterials provide a flexible platform for modulating OAM.

In the considered nanoantenna array metamaterial system, the symmetrical characteristic of the meta-atom is crucial for effective generation of OAM entangled photons through PDC process. For the first point, the available order of nonlinear susceptibility directly depends on the symmetry of the nanoantenna. A selection rule can be summarized for the harmonic generation of circular polarized waves in the corresponding system. When the meta-atoms possess an *m*-fold rotational symmetry, the harmonic order *n* should satisfy the condition *n* = *jm* ± 1, where *j* is an integer to be determined [34]. The sign ‘+’ and ‘−’ correspond to the situations in which the fundamental and harmonic waves have the same and opposite circular polarizations, respectively. Correspondingly, the *n*th-order nonlinear polarization can be expressed as [35]:

(3) $$P_{\theta ,\;\pm \sigma }^{\left(n\right)}\propto e^{i(n\;\mp \;1)\sigma \theta }{\left(E^{\sigma }\right)}^{n}$$

From the equation above, it can be seen that an additional geometric phase $e^{i(n\;\mp \;1)\sigma \theta }$ emerges, which is related to the orientation *θ* of the meta-atom. Thus, the spatial properties of the photon states can be tailored through arranging the meta-atoms. To introduce OAM into the system, *θ* is defined as *qφ*(*x*, *y*), where *φ*(*x*, *y*) = arctan(*x*, *y*) and *q* is a variable geometry parameter. To clarify the evolution rule of OAM, we shall consider the harmonic generation process first. If the fundamental pump light has a circular polarization state of *σ*, the *n*th harmonic wave acquires an OAM of l=(n ∓ 1)σq with the helicity ±*σ*. In the conjugated parametric process, this rule still holds.

**Figure 2:**
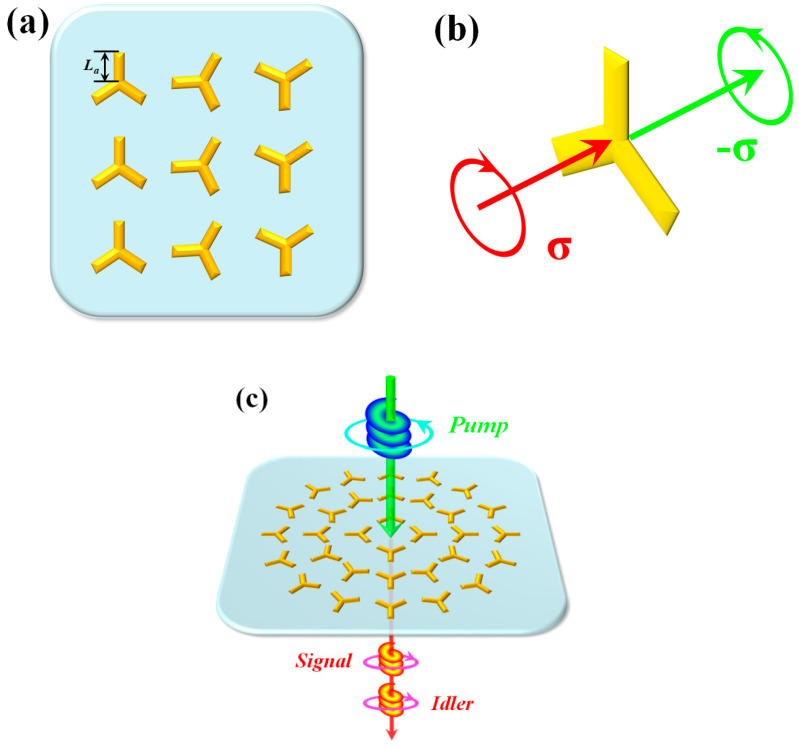
(**a**) The C3 nanoantenna array system. The length of meta-atom arm is marked as *L_a_*. (**b**) The quadratic interaction with C3 rotationally symmetrical meta-atom. (**c**) The mechanism of PDC process in the C3 nonlinear metamaterial system.

For PDC, the harmonic order is *n* = 2, thus the only available case is *j* = 1 & *m* = 3 with the sign ‘−’ according to the selection rule. Therefore, we consider the 3-fold (C3) rotationally symmetrical meta-atom for the present work, as is shown in Figure 2a. The nonlinear polarization is $P_{\theta ,\;-\sigma }^{\left(2\right)}=\chi_{max}^{\left(2\right)}e^{i3\sigma \theta }E_{1}^{\sigma }E_{2}^{\sigma }$. In this equation, $\chi_{max}^{\left(n\right)}$ represents the maximum of effective nonlinear coefficient, while the actual local value depends on the geometric phase *e^i3σθ^* which is related to the orientation *θ* of the C3 meta-atom. The illustration of the corresponding quadratic interaction is shown in Figure 2b. As regards the OAM, under the evolution rule, the topological charge should be *l* = 3*σq*. Thus, the PDC process can be expressed as $p_{l_{p},\;\sigma }\to s_{l_{s},\;-\sigma }+i_{l_{i},\;-\sigma }$, where *l_s_* + *l_i_* = *l_p_* + 3*σq*. The schematic is given in Figure 2c.

For effective generation of the OAM entangled state, the orientation distribution of the C3 meta-atoms should be further regulated. Besides the evolution of OAM, there is another aspect to be considered. That is the issue of linear-momentum conservation, which is crucial for a high transformation efficiency of the PDC process. To ensure this point, the pump light should be obliquely launched, and the transverse mismatch of wave vector can be compensated by the gradient of meta-atom orientation. Therefore, additional phase modulation is introduced as Ψ_1_(*ψ*) = (*dψ*/*dx*)·*x* besides the Ψ_0_(*φ*) = 3*σqφ*. Thus the total distribution function of the orientation angle is derived as:

(4) $$\theta =q\varphi +\frac{(d\psi /dx)\cdot x}{3\sigma }$$

**Figure 3:**
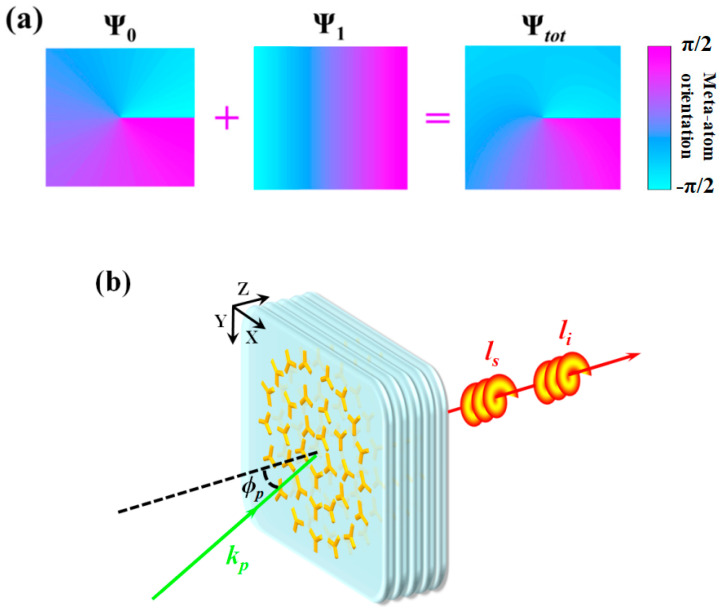
(**a**) Constructing the total distribution function Ψ*_tot_* of meta-atom orientation. (**b**) The principle of PDC process in the nonlinear metamaterial with the distribution Ψ*_tot_*. The pump light is obliquely launched to ensure a high transformation efficiency.

If the wavelength is set at 0.55 μm and the input angle is 3°, the equivalent transverse wave vector *k_NM_* = *dψ*/*dx* is calculated to be 0.60 μm^−1^ (*k_NM_* = *k_pt_* = 2π/0.55 × sin(3°)). Together with the conditions *σ* = 1 and *q* = 1/3, we present an illustration of the corresponding distribution of orientation angle in Figure 3a.

To further clarify the properties of OAM entangled state generated in the proposed system, we derive the two-photon state vector. As the conservation of longitudinal linear momentum has been satisfied, it is not necessary to include *z*-dependent variation of $\chi_{NM}^{\left(2\right)}$. Hence, we can take identical 2D slices to stack for 3D metamaterials. If the two down-converted photons are degenerate, the influences of the intrinsic loss of nonlinear metamaterials act on them equally. As a result, the attenuation does not affect the quantum properties of the OAM entangled state and only draw down the generation efficiency. Correspondingly, the situation is further simplified, and the visibility in the coincidence measurement of signal and idler photons still maintains. We choose the pump light as *E_p_*$\left(\vec{r}\right)$ = *E*_*p*0_exp(*ik_p_*·$\vec{r}$ + *il_p_φ*), and the transverse profiles Ψ*_st_*$\left(\vec{r}\right)$ and Ψ*_it_*$\left(\vec{r}\right)$ of signal and idler photons are supposed to be exp(*il_s_φ*) and exp(*il_i_φ*). The input angle of the pump light is *ϕ_p_*. With all these expressions, we can obtain the form of state vector as:

(5) $$\begin{array}{cc} |\Psi \rangle =|0\rangle & +\frac{iE_{p0}\chi_{max}^{\left(2\right)}}{4\hbar }\int d{\vec{k}}_{s}\int d{\vec{k}}_{i}\int d\vec{r}\\ & \times F_{s,\;-\sigma }F_{i,\;-\sigma }e^{i(d\psi /dx)}e^{i({\vec{k}}_{p}-{\vec{k}}_{s}-{\vec{k}}_{i})\;\cdot \;\vec{r}}\\ & \times e^{i(l_{p}+3\sigma q-l_{s}-l_{i})\varphi }a_{s}^{†}a_{i}^{†}|0\rangle \end{array}$$

According to this equation, to eliminate the phase term $e^{i(d\psi /dx)}e^{i({\vec{k}}_{p}-{\vec{k}}_{s}-{\vec{k}}_{i})\cdot \vec{r}}$, the propagation directions of the signal and idler photons are considered to be along the *z*-axis, thus the input angle *ϕ_p_* should satisfy the condition: *k_p_*sin*ϕ_p_* = *dψ*/*dx* and *k_p_*cos*ϕ_p_* = *k_s_* + *k_i_*. As is shown in Figure 3b, when the pump light is obliquely launched along the positive *x*-axis, the OAM satisfies *l_s_* + *l_i_* = *l_p_* + *3σq*, and the state vector can be reduced to $|\Psi \rangle =\sum F_{l_{s},\;l_{i}}|l_{s}\rangle |l_{i}\rangle$. As the azimuthal modes {|l〉} are automatic Schmidt eigenvectors [36], *F_ls,li_* should be the corresponding Schmidt eigenvalue, which can be derived through Schmidt decomposition [37].

**Figure 4:**
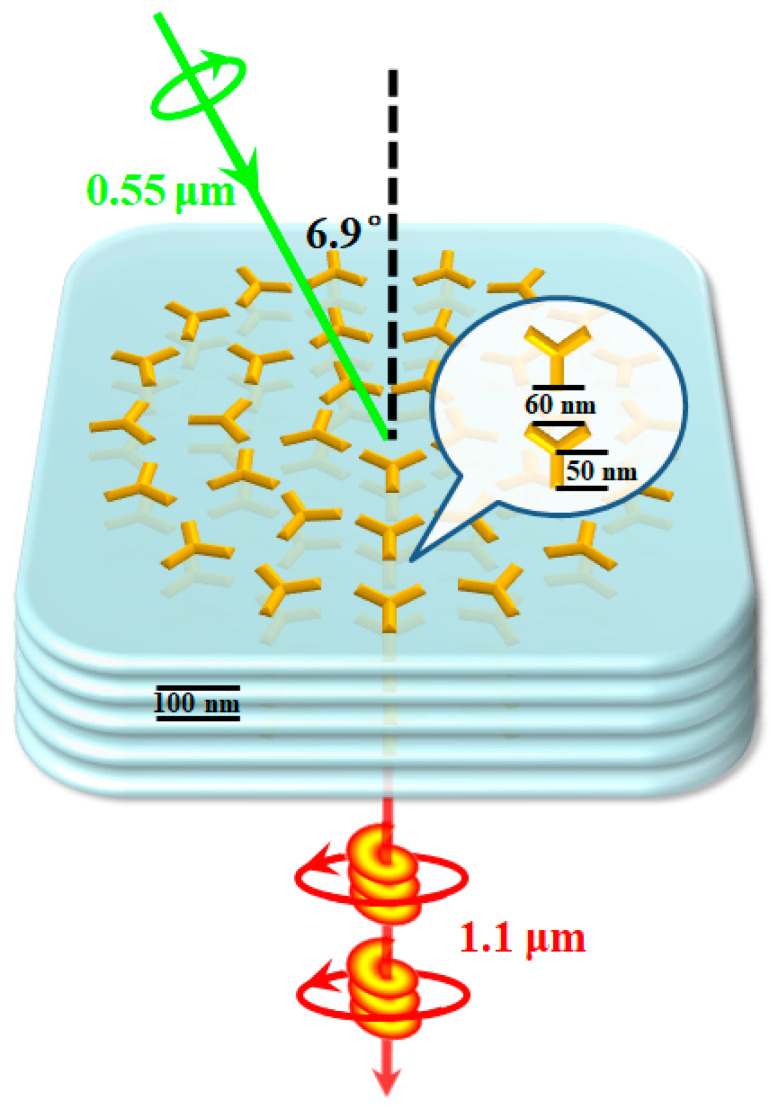
The metamaterial design. The thickness of nanoantenna is 40 nm, while the gradient of meta-atom orientation along the *x*-direction is *dψ*/*dx* = 1.83 × 10^6^ m^−1^. The pump light is in right-hand circular polarization, thus the down-converted photons are in left-hand circular polarization.

**Figure 5:**
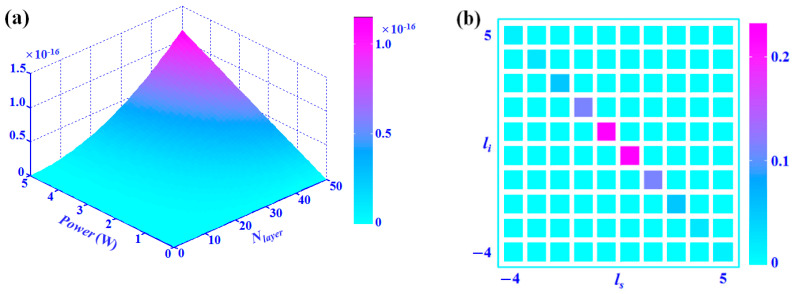
(**a**) The generation efficiency of entangled states for the case (*l_p_* = 0 & *q* = 1/3) corresponding to varying pump power and layer number. (**b**) The coincidence probabilities as a function of *l_s_* and *l_i_* for (*l_p_* = 0; *q* = 1/3).

For a concrete illustration, we do further calculations based on an actual system. The material of meta-atoms is chosen to be gold, and the silica substrate is considered. The geometry options are as follows: the length *L_a_* of meta-atom arm is 50 nm, and the distance between two meta-atoms along the radial direction is 60 nm. In addition, the thickness of gold is 40 nm, while the effective thickness of one layer in the metamaterial is ~100 nm. Moreover, the pump wavelength is set at 0.55 μm, while the signal and idler wavelengths are set to degenerate at 1.1 μm. Theoretically, in such a system, there are issues of reflection and absorption for the photons in each metamaterial layer, which seriously hinders the accumulation of PDC effect. However, according to Ref. [24], this problem can be dealt with based on small meta-atoms which are not densely packed. Hence, we only consider the attenuation of signal and idler photons caused by metallic components of the metamaterials. The corresponding effective attenuation rate is estimated to be *α_s/i_* = 1.71 × 10^4^ m^−1^. In such cases, the input angle should be *ϕ_p_* = 6.9°, thus the gradient of meta-atom orientation along the *x*-direction is *dψ*/*dx* = 1.83 × 10^6^ m^−1^. While the pump light is right-circular polarized, the down-converted photons are in the left-circular polarization state. Figure 4 is illustrated to clarify the design. The quadratic nonlinear susceptibility $\chi_{max}^{\left(2\right)}$ is in the magnitude of 10^1^ pm/V [14,20], thus the generation efficiency of entangled states for the case (*l_p_* = 0 & *q* = 1/3) is calculated according to varying pump power and layer number. The results are shown in Figure 5a. In actual systems, the effective susceptibility might be higher, and a femtosecond laser can be chosen as the pump source to enhance the emission. If the laser parameters are in the magnitude of 10^1^ μJ/pulse and 10^2^ fs, the production rate of photon-pair can reach above 10^−2^ per pulse, which is available for practical applications. To further reveal the quantum properties of the generated OAM entangled state, the coincidence probabilities |*F_ls_*_,*li*_|^2^ are plotted as a function of *l_s_* and *l_i_* for (*l_p_* = 0; *q* = 1/3) in Figure 5b.

Based on the above discussions, it can be seen that nonlinear metamaterials provide an effective platform for tuning the spatial properties of entangled photons. To consider the realization of a practical experimental system, metamaterials with 10 several layers can be achieved based on electron beam lithography [38]. For more layers, improved technologies, such as femtosecond laser-induced forward-transfer technique [39], bring opportunities, and cascading metamaterial samples also provides a possible avenue. Another thought is using nonlinear materials with larger *χ*^(2)^, which can reduce the number of layers necessary for fabrication. The multi-quantum-wells provide an ideal choice [40,41,42]. The coincidence measurement can still be utilized for presenting entanglement [43]. Besides the OAM entanglement, entangled photon states of more complex special spatial modes, such as Hermite-Gaussian modes may also be realized with suitable design of the metamaterials.

Practical quantum applications require multipartite systems. Traditionally, multi-photon entanglement is explored for this target. However, the corresponding generation efficiency decreases dramatically along with the rising of photon number [44], which brings enormous challenges. Changing the thought, the multi-dimensional entanglement can be chosen as an alternative in certain situations, and the optical OAM system is a representative one. The abundant nanostructures of metamaterials just provide a powerful tool to establish various “nanostructured” fields through controlling light–matter interaction. The rapidly developing processing technologies make the system highly designable, which ensure the capacity of devising the entangled photons.

## 4. Conclusions

We have investigated tailoring photonic spatial entanglement based on the PDC process in the nonlinear metamaterial consisting of C3 rotationally symmetrical meta-atoms. Compared with the previously demonstrated lithium niobate and semiconductor metasurfaces without considering spatial entanglement, we are devoted to exploring the potential of OAM degree of freedom. Through suitable nanostructure design, the OAM entangled photons can be effectively obtained. The theoretical framework is carried out based on the combination of nonlinear Huygens–Fresnel principle and propagation damping theories. Overall, the nonlinear metamaterials provide a flexible platform for devising photonic entanglement, and this platform can be further extended for significant applications, such as quantum interference and quantum imaging.

## Data Availability

The data presented in this study are available on request from the corresponding author.
